# Bioengineering of LAB vector expressing Haemolysin co-regulated protein (Hcp): a strategic approach to control gut colonization of *Campylobacter jejuni* in a murine model

**DOI:** 10.1186/s13099-021-00444-2

**Published:** 2021-07-30

**Authors:** Chandan Gorain, Afruja Khan, Ankita Singh, Samiran Mondal, Amirul Islam Mallick

**Affiliations:** 1 Department of Biological Sciences, Indian Institute of Science Education and Research Kolkata, Mohanpur, Nadia, West Bengal 741246 India; 2 Department of Veterinary Pathology, West Bengal University of Animal and Fishery Sciences, Belgachia, Kolkata, West Bengal 700037 India

**Keywords:** *Campylobacter jejuni*, Haemolysin co-regulated protein, Lactic acid bacteria (LAB), Mucosal vaccine, Immune-protection

## Abstract

**Background:**

*Campylobacter jejuni* (*C. jejuni*) is accountable for more than 400 million cases of gastroenteritis each year and is listed as a high-priority gut pathogen by the World Health Organization (WHO). Although the acute infection of *C. jejuni* (campylobacteriosis) is commonly treated with macrolides and fluoroquinolones, the emergence of antibiotic resistance among *C. jejuni* warrants the need for an alternative approach to control campylobacteriosis in humans. To this end, vaccines remain a safe, effective, and widely accepted strategy for controlling emerging and re-emerging infectious diseases. In search of a suitable vaccine against campylobacteriosis, recently, we demonstrated the potential of recombinant Haemolysin co-regulated protein (Hcp) of *C. jejuni* Type VI secretion system (T6SS) in imparting significant immune-protection against cecal colonization of *C. jejuni;* however, in the avian model. Since clinical features of human campylobacteriosis are more complicated than the avians, we explored the potential of Hcp as a T6SS targeted vaccine in a murine model as a more reliable and reproducible experimental host to study vaccine-induced immune-protection against *C. jejuni*. Because *C. jejuni* primarily utilizes the mucosal route for host pathogenesis, we analyzed the immunogenicity of a mucosally deliverable bioengineered Lactic acid bacteria (LAB), *Lactococcus lactis* (*L. lactis*), expressing Hcp. Considering the role of Hcp in both structural (membrane-bound) and functional (effector protein) exhibition of *C. jejuni* T6SS, a head-to-head comparison of two different forms of recombinant LAB vectors (cell wall anchored and secreted form of Hcp) were tested and assessed for the immune phenotypes of each modality in BALB/c mice.

**Results:**

We show that regardless of the Hcp protein localization, mucosal delivery of bioengineered LAB vector expressing Hcp induced high-level production of antigen-specific neutralizing antibody (sIgA) in the gut with the potential to reduce the cecal load of *C. jejuni* in mice.

**Conclusion:**

Together with the non-commensal nature of *L. lactis,* short gut transit time in humans, and the ability to express the heterologous protein in the gut, the present study highlights the benefits of bioengineered LAB vectors based mucosal vaccine modality against *C. jejuni* without the risk of immunotolerance.

**Supplementary Information:**

The online version contains supplementary material available at 10.1186/s13099-021-00444-2.

## Background

Acute gastroenteritis of bacterial origin is accountable for nearly 78% of diarrhoeal diseases in low and middle-income countries (LMIC) [1–3]. Children under five experience nearly 1.4 billion episodes of acute diarrhoea each year, with approximately two million deaths annually [4]. The most common cause for diarrhoea associated illness includes infection of *Escherichia coli* (*E. coli*), *Vibrio cholerae*, *Campylobacter* spp., *Salmonella* spp., *Aeromonas* spp., and *Yersinia enterocolitica* [5–7]. Worldwide, *Campylobacter jejuni* (*C. jejuni*) is accountable for more than 400 million cases of gastroenteritis each year and is listed as a high-priority gut pathogen by the World Health Organization (WHO) [8–10]. Growing evidence of clinical studies also suggests the role of *C. jejuni* infection with several extra-intestinal complications, including Guillain–Barre syndrome (GBS), reactive arthritis (RA), Miller Fisher syndrome (MFS), and irritable bowel syndrome (IBS) [11–14].

As a self-limited disease, *Campylobacter*-associated diarrhoeal illness (campylobacteriosis) are commonly treated with antibiotics, particularly with primary fluoroquinolones and macrolides. However, rapid emergence of antibiotic resistance among *C. jejuni* strains has been reported from several countries, raising serious concerns worldwide [15–17]. Although campylobacteriosis is a vaccine-preventable disease, no licensed vaccine is available for the humans or other hosts. Over the last twenty years, several experimental vaccines against *C. jejuni* have been tested; among them, live attenuated, killed whole-cell, subunit, capsule-conjugated and glycoconjugated vaccines are of note [18]. However, limited understanding of *C. jejuni* pathogenesis, structural variation in capsular polysaccharides (CPS), and importantly, the risk of autoimmune disorder due to gangliosides mimic epitopes in bacterial lipooligosaccharides (LOS) emerged as the most outstanding issues for a successful vaccine against *C. jejuni* [11, 18]. Moreover, the lack of a pertinent animal model to test the vaccine-induced immune-protection remains another bottleneck for *C. jejuni* vaccine. Thus, developing an effective measure to control *C. jejuni* infection in humans demands a critical understanding of the molecular basis of *C. jejuni* pathogenesis and the introduction of logical approaches that can improvise more empirical strategies used in the past.

As a key player in the intestine, the gut-associated lymphoid tissues (GALTs) exhibit a complex network to regulate cellular and molecular events in response to gut pathogens [19]. The ability to discriminate the commensal and pathogenic invader by GALTs is primarily controlled by the tolerogenic environment of the intestinal tract, which often poses additional challenges for the mucosal vaccine efficacy. Therefore, the success of a vaccine against common gut pathogens largely relies on the nature of the immune responses during infection, selection of the target antigens, dose determination as well as appropriate mode of antigen delivery [20].

To this end, mucosal delivery of several surface-exposed colonization proteins (SECPs), including Jejuni lipoprotein A (JlpA), *Campylobacter* adhesion of fibronectin (CadF), Fibronectin like protein A (FlpA) and other outer membrane proteins (OMPs) have been explored in the past using a range of delivery mode including several biodegradable polymers such as liposomes, nanoparticles, and microneedles [21–24].

Recently, the bacterial secretion system (Type I-IX) as multi-component secretion machinery has emerged as an attractive target for identifying small molecules, peptides, and monoclonal antibodies based strategy to restrict the secretion system associated virulence [25–27]. Among the 13 genes that encode a functional T6SS, Haemolysin co-regulated protein (Hcp) is considered as a key effector protein that facilitates bacterial adherence and cell cytotoxicity [28, 29]. In addition to T6SS functionality, regardless of serotypes, Hcp protein is also essential for both T6SS assembly and effector function [30]. Our recent in silico study also suggests several conserved putative B cell epitopes in the Hcp sequence of *C. jejuni* [31]. Therefore, effective neutralization of such a critical virulence elements Hcp would minimize the host pathogenicity by neutralizing toxins, blocking bacterial attachment and clearance of a wide range of high pathogenic *C. jejuni* strains. In view of the multifaceted function of Hcp, our group has constantly pursued the possible application of Hcp as a T6SS targeted vaccine.

In the present study, we chose to extrapolate the benefit of using Hcp as a potential vaccine candidate for humans using mice model. Moreover, to deliver the target protein directly at the host–pathogen interface, we used a food-grade Lactic acid bacterium (LAB), *Lactococcus lactis* (*L. lactis*) as a mucosal delivery platform. Considering the risk of immune tolerance due to unregulated expression of target protein at the mucosal surface, we used NIsin Controlled gene Expression (NICE) system to permit regulated expression of Hcp in the gut [32]. Unlike other Gram-negative bacteria, *C. jejuni* T6SS possesses only one type of gene purported to play structural and effector functions. Hence, two different forms of LAB vectors were bioengineered to express Hcp, one as secreted (Sec-Hcp) and the other as cell wall anchored form (CWA-Hcp). Here, we provide direct evidence that mucosal delivery of both forms of Hcp expressing r*L. lactis* in mice can elicit a significantly higher level of functional secretory IgA (sIgA) responses and block *C. jejuni* colonization. Analysis of immunological correlates of protection further suggests the ability of the present vaccine modality in protecting gut mucosa against *C. jejuni* challenge.

## Methods

### Bacterial strains, plasmids, and culture conditions

Bacterial strains and plasmids used in this study are listed in Table 1. A human isolate of *C. jejuni* (BCH71) used in this study was kindly provided by Dr. Asish Kumar Mukhopadhyay, Scientist F, NICED (ICMR), Kolkata, India, and maintained in our laboratory as per the standard method [23]. *E. coli* (DH5α, Top 10 and M15) and *L. lactis* subsp. *cremoris* MG1363 (NZ9000) strains were used for gene cloning and protein expression study. *L. lactis* strains were regularly grown at 30 °C in M17 medium (HiMedia, India) containing 0.5% (w/v) glucose (GM17) supplemented with chloramphenicol (20 µg/mL) without agitation. *C. jejuni* isolate was grown in Mueller–Hinton (MH) medium (HiMedia) having CAT (cefoperazone 8 mg/L, amphotericin 10 mg/L, and teicoplanin 4 mg/L; HiMedia) selective supplement at 37 °C under microaerophilic conditions (10% CO_2_, 5% O_2_, and 85% N_2_) in a tri-gas incubator (Thermo Fisher Scientific, USA). *E. coli* strains were grown in Luria–Bertani (LB) medium (HiMedia) at 37 °C supplemented with ampicillin (50 µg/mL) and/or kanamycin (25 µg/mL).

**Table 1:** Bacterial strains and plasmids used in this study

Bacterial strains and plasmids	Characteristics	Purpose	Source
Strains			
* E. coli* M15	F-, Φ80ΔlacM15, thi, lac-, mtl-, recA + , KmR	Recombinant protein expression (pQE30-*hcp*)	Qiagen, USA
* E. coli* DH5α	F-u80dlacZDM15 D (lacZYA- argF) U169 endA1 recA1 hsdR17(rk- mk) deoR thi-1 supE44 k-gyrA96 relA1	Recombinant plasmid storage (cloning vector)	BioBharati, India
* E. coli* Top 10	F- mcrA Δ(mrr-hsdRMS-mcrBC) Φ80lacZΔM15 Δ lacX74 recA1 araD139 Δ(araleu)7697 galU galK rpsL (StrR) endA1 nupG	Recombinant plasmid storage (*L. lactis* based plasmid constructs)	Thermo Fisher Scientific, USA
* L. lactis* NZ9000	MG1363 (nisRK genes into chromosome), Wild type, plasmid free	Wild Type Control	Dr. L. G. Bermúdez Humarán, INRA, France
* C. jejuni*	Human clinical isolate (BCH 71)	For *hcp* gene cloning and infection study	Dr. Asish Kumar Mukhopadhyay, Scientist F, NICED (ICMR), Kolkata, India
Plasmids			
pQE30	Amp^r^, His6 (N terminal), T5 promoter	Prokaryotic expression vector	Addgene, USA
pQE30-*hcp*	Amp^r^, pQE30 harboring *hcp*	Recombinant Hcp expression vector	[31]
pNZ8048-CWA_M6_	Cm^r^, spUSP45, M6 cell wall anchor expressed under P_*nisA*_ promoter	*L. lactis* based rHcp expression vector	Dr. L. G. Bermúdez Humarán, INRA, France
pNZ8048-CWA_M6-_ *hcp*	Cm^r^, spUSP45, M6 cell wall anchor expressed under P_*nisA*_ promoter	*L. lactis* based rHcp expression vector	This work
pSEC-*hcp*	Cm^r^, spUSP45 under P_*nisA*_ promoter	*L. lactis* based rHcp expression vector	This work

### Plasmid construction for Hcp expression by bioengineered LAB vector

#### Generation of recombinant LAB (rLAB) vector surface expressing Hcp (CWA-Hcp)

**Fig. 1:**
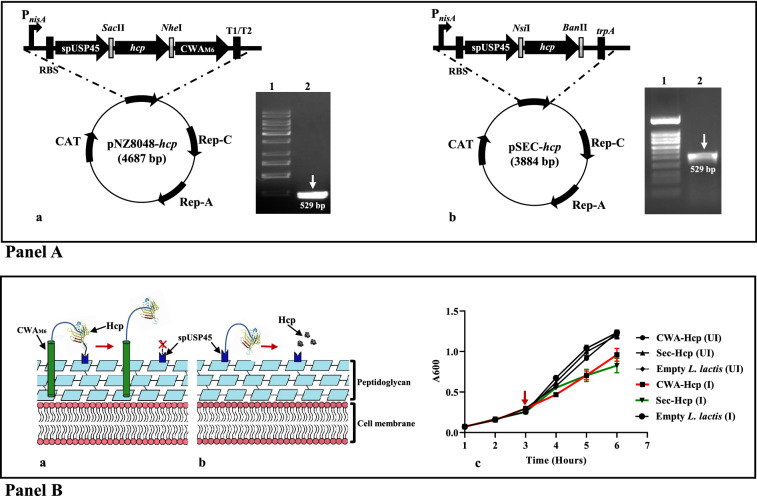
Construction of nisin inducible r*L. lactis* vector expressing Hcp protein. **A** Schematic of NICE cassette of pNZ8048-spUSP45-*hcp-*CWA_M6_ plasmid under P_*nisA*_ promoter for a surface-anchored form of Hcp. Colony PCR of positive transformant of r*L. lactis* cells harbouring *hcp* gene showing amplification at the expected size (~ 529 bp) (**a**). Schematic of r*L. lactis* having pSEC-spUSP45-*hcp* plasmid construct without CWA_M6_ cell wall anchor motif. Colony PCR of positive transformant of r*L. Lactis* harbouring *hcp* gene insert showing the amplified product at the expected size (~ 529 bp) (**b**). **B** Graphical depiction of surface display of Hcp protein anchored to the cell wall of r*L. lactis* via cell wall anchoring motif of M6 (CWA_M6_) protein of *Streptococcus pyogenes* (**a**). Graphical depiction of r*L. lactis* secreting Hcp in the extracellular matrix (**b**). Comparative growth profile of r*L. lactis* harbouring pNZ8048-spUSP45-*hcp-*CWA_M6_ and pSEC-spUSP45-*hcp* plasmid showing altered (reduced) growth kinetics of nisin-induced r*L. lactis* compared to empty *L. lactis* (NZ9000) and un-induced r*L. lactis* cells. Arrow indicates the time of nisin induction (**c**)

The coding sequence of *hcp* gene (517 bp) was PCR amplified from previously constructed pQE30-*hcp* plasmid using specific primer sets (Table 2) and cloned into a pNZ8048 based plasmid backbone between the N-terminal signal peptide (SP) of *L. lactis* protease USP45 (spUSP45; 81 bp) and C-terminal of a cell wall anchored (CWA_M6_; 424 bp) domain of *Streptococcus pyogenes* (*S. pyogenes*) M6 protein between the restriction sites *Sac*II and *Nhe*I. NICE system driven by P_*nisA*_ promoter was used for the expression of the target protein. The newly constructed recombinant plasmid was designated as pNZ8048-*hcp* (Fig. 1A-a). Next, the recombinant pNZ8048-*hcp* plasmid was electro-transformed into electro-competent *L. lactis* NZ9000 cells using GenePulser (Bio-Rad, USA; 2.0 kV, 25 µF and 200 Ω) and grown on GM17 agar medium supplemented with chloramphenicol. Positive transformants were screened by colony PCR followed by sequence analysis. A graphic depiction of surface display of fusion protein expressed by r*L. lactis* cells is presented in Fig. 1B-a.

**Table 2:** List of primers used in this study

Target Gene	Primer Sequence (5′-3′)	Amplicon size (bp)	Ref
pQE30-*hcp*	F-5′ CCGCGGTACCATGG CTGAACCAGCGTTTATAAAAATTG 3′ R-5′ GACTACTGCAGTT AAGCTTTGCCCTCTCTCCA 3′	510	[31]
pNZ8048-*hcp*	F-5′ CATCCGCGGATGGCTGAACCAGCGTTT 3′ R-5′ GATGCTAGCGAGCTTTGCCCTCTCTCCA 3′	529	This work
pSEC-*hcp*	F-5′ CGCATGCATATGGCTGAACCAGCGTTTATA 3′ R-5′ CAGGAGCTCTTA AAGCTTTGCCCTCTCTCC 3′	529	This work

#### Generation of rLAB vector secreting Hcp (Sec-Hcp)

To generate rLAB vector expressing the secretory form of Hcp, we used pSEC backbone of a Lactococcal vector to clone *hcp* gene at the C-terminal of spUSP45 sequence between the restriction site *Nsi*I and *Ban*II followed by electro transformation into *L. lactis* NZ9000 cells. (Fig. 1A-b). A graphic depiction of r*L. lactis* secreting Hcp is shown in Fig. 1B-b.

#### Optimization of protein expression by rLAB vectors

Recombinant *L. lactis* cells harbouring either pNZ8048-*hcp* or pSEC-*hcp* plasmid were grown in GM17 medium at 30 °C without agitation. To optimize the protein expression, varied concentrations of nisin (5–15 ng/mL) were used when OD_600_ reached 0.3. Growth kinetics of un-induced (UI) and nisin-induced (I) r*L. lactis* cells were monitored till 6 h to determine the optimum concentration of nisin required for Hcp expression.

#### Detection of surface expression of Hcp by induced rLAB vector (CWA-Hcp)

*Immunofluorescence microscopy*: Immunofluorescence microscopy was performed to assess the ability of rLAB vector to express Hcp on the cell surface. Briefly, 1 × 10^9^ r*L. lactis* cells were induced with nisin (15 ng/mL) and harvested at 4 h post-induction. Cells were washed with PBS followed by fixation with pre-chilled 4% paraformaldehyde (PFA) (Sigma, USA) for 30 min on ice. After thorough washing, cells were blocked with 3% BSA (HiMedia) for 1 h at room temperature (RT). Rabbit polyclonal anti-Hcp antibody was used as primary antibody (1:50 dilution), and FITC conjugated goat anti-rabbit IgG (H + L) (Thermo Fisher Scientific) was used as the secondary antibody (1:500 dilution). Finally, the washed cells were mounted with 5 µL of Vectashield mounting media (Vector Laboratories, USA) on a glass slide, and images were captured in Olympus 1X epifluorescence microscope equipped with FITC filter in 60X magnification with an excitation wavelength of 455–495 nm and emission wavelength of 505–555 nm range. Empty NZ9000 cells (without recombinant plasmid) and un-induced *rL. lactis* cells were kept as control.

*Flow cytometric analysis*: To further analyze the surface expression of rHcp, flow cytometry was performed. Recombinant *L. lactis* cells were processed as per the method described in the above section, except the fixation step which was performed after antibody staining. Finally, the cells were analyzed by BD FACSCalibur flow cytometer using FITC filter in FL1 channel (Excitation wavelength: 488 nm and Emission wavelength: 530 nm). Unstained r*L. lactis,* empty NZ9000 cells, and un-induced r*L. lactis* cells were kept as control.

#### Detection of Hcp secreted by rLAB vector (Sec-Hcp)

*Indirect ELISA*: To detect Hcp secreted in the culture medium of nisin-induced r*L. lactis* cells, indirect ELISA was performed as described previously with some modifications [33]. Briefly, supernatant of r*L. lactis* at 4 h post-induction was collected, and total protein was precipitated by trichloroacetic acid (TCA) (Sigma). The precipitates thus formed were washed with chilled acetone and dried to remove acetone. Finally, protein pellets were resuspended in PBS and coated (using carbonate bicarbonate buffer, pH-9.6) in 96 well ELISA plate (Nunc, USA) overnight at 4 °C. Next, the plate was thoroughly washed with PBS-T (0.05% Tween-20 in PBS) and blocked with PBS-T containing 5% BSA for 1 h at 37 °C. Following, 100 μL of rabbit polyclonal anti-Hcp antibody (1:500 dilution) was added to each well and kept for 2 h at RT. After thorough washes, horseradish peroxidase (HRP) conjugated goat anti-rabbit IgG (H + L) secondary antibody (1:2500 dilution) (Biobharati, India) was added to each well and incubated for another 1 h at RT. Subsequently, wells were treated with TMB (3,3′,5,5′-tetramethylbenzidine) (HiMedia) substrate, and the reaction was stopped by adding 50 µL of stop solution (1M sulphuric acid) (Merck, USA). The absorbance was read at 450 nm in a microplate reader (BioTek, USA). Purified rHcp protein expressed by *E. coli* and native Hcp secreted by our laboratory isolate of *C. jejuni* (18aM) were kept as a positive control.

*Dot blot analysis*: For dot blot analysis, the culture supernatant was processed as described in the previous section and spotted onto polyvinylidene fluoride membrane (PVDF) (Merck). Further, the membrane was blocked with 3% BSA in 50 mM Tris–HCl buffer (pH 7.4) containing 0.05% Tween 20 (TBS-T) (BR BIOCHEM, India) for 1 h at 4 °C. The blocked membrane was then washed five times (2 times with TBS-T and 3 times with TBS) and incubated with rabbit polyclonal anti-Hcp antibody (1:500 dilution) for 1 h at RT. Next, the membrane was washed and incubated with HRP conjugated goat anti-rabbit IgG (H + L) secondary antibody (1: 2500 dilution) for 1 h at RT. The blot was developed using 3,3′-Diaminobenzidine (DAB) (Sigma) as a substrate.

### In vivo mice immunization and immuno-protection study

#### Preparation of rLAB vector for mice immunization

For oral administration in mice, both forms of r*L. lactis* (cell wall anchored and secreted form) cells were induced with 15 ng/mL of nisin when OD_600_ reached 0.3. At 4 h post-induction, bacterial cells were harvested by centrifugation at 6000×*g* and washed with PBS. Finally, the cell number was adjusted to 2 × 10^9^ in 100 µL of PBS containing 0.5% (w/v) glucose.

#### Mice immunization and sample collection

A total of 40 female 6-week-old BALB/c mice were kept at 24 °C with 12 h day-night cycles and provided food and water ad libitum. Vaccination was performed using a prime-boost strategy. Mice were randomly separated into four experimental groups with ten mice (n = 10) in each and orally administered through oral gavage with the following treatments: Group A: PBS only; Group B: 2 × 10^9^ CFU of empty NZ9000 cells; Group C: 2 × 10^9^ CFU of r*L. lactis* expressing CWA-Hcp; Group D: 2 × 10^9^ r*L. lactis* expressing Sec-Hcp. Details of the mice immunization schedule are provided in Fig. 3a.

Fresh fecal pellets were collected from individual mice of each group at day 7 post last immunization and mixed with 200 µL of IgA extraction buffer (PBS containing 0.5% Tween 20, 1 mg/mL EDTA and protease inhibitors). Pellets were vigorously vortexed, followed by centrifugation at 10,000×*g* for 10 min. The supernatant was collected and kept at − 20 °C for further use. Blood samples were collected by retro-orbital puncture and centrifuged at 1000×*g* for 10 min at 4 °C to collect the sera and stored at − 20 °C.

Three mice from each group were sacrificed by cervical dislocation to collect spleen for splenocyte proliferation assay on day 7 post last immunization (day 28), while the remaining mice were orally challenged with 100 µL of 2 × 10^9^ CFU of *C. jejuni* (BCH71)*.*

Finally, challenged mice were sacrificed on day 7 post-infection (day 35), and cecal tissues and their contents were collected for histopathological analysis and determining the bacterial load.

#### Assessment of local (sIgA) and systemic (IgG) antibody responses

Fecal soups or sera samples collected from the mice belonging to different experimental groups were subjected to indirect ELISA to detect Hcp-specific antibody response as per published methods [34]. Briefly, ELISA plates were coated with 100 ng/well of *E. coli* expressed rHcp protein prepared in coating buffer (carbonate bicarbonate buffer pH-9.6) overnight at 4 °C. The next day, plates were washed with PBS and blocked with 5% BSA for 1 h at 37 °C. After thorough washing of the wells, serially diluted fecal soups or sera samples were added to each well and incubated for 2 h. Plates were then washed with PBS and probed with anti-Hcp antibody as primary and HRP-conjugated goat anti-mouse IgA or IgG as the secondary antibody (1: 2500 dilution; Thermo Fisher Scientific) for 1 h at RT. Following three washes with PBS, TMB substrate was added to each well, and the reaction was stopped with 50 µL of stop solution (1 M H_2_SO_4_). Finally, the absorbance was read at 450 nm in a microplate reader (BioTek).

#### Assessment of cellular responses

*In vitro splenocyte proliferation assay*: Spleen from each experimental mice (n = 6) was collected, and single-cell suspension was prepared as per the method described elsewhere [35]. Briefly, the spleens were teased against a disposable syringe head in a sterile petriplate containing RPMI 1640 medium (Gibco, USA). The cell suspension was aspirated and filtered through a cell strainer (70 µm) (Genetix Biotech, India). The filtrate containing the single-cell suspension was layered onto pre-warmed Histopaque solution (Sigma) in 1:1 ratio and centrifuged at 500 ×*g* for 20 min at RT. The interface containing splenocytes was collected, washed and resuspended in a complete growth medium (RPMI 1640). Subsequently, the splenocyte proliferation assay was performed following the standard method with slight modifications [36]. Briefly, 2 × 10^5^ cells in RPMI 1640 medium were added to each well of a 96-well tissue culture plate in triplicates. After 3 h, cells were stimulated with 10 µg/mL of rHcp protein expressed and purified from *E. coli*. In contrast, unstimulated splenocytes and splenocytes stimulated with concanavalin A (Con A) (10 µg/mL) were kept as controls. After 24 h of incubation, 10 μL of MTT (3-[4, 5-dimethylthiazolyl-2]-2, 5-diphenyltetrazoliumbromide) (1 mg/mL) was added to the corresponding wells and incubated for additional 3 h at 37 °C under 5% CO_2_ pressure. The insoluble formazan crystals formed were solubilized with DMSO (dimethyl sulphoxide) (Merck), and the absorbance was recorded at 595 nm using a microplate reader. For each experimental group, splenic lymphocyte proliferation index (Stimulation Index; SI) was calculated as follows:

Stimulation Index (SI) = Mean absorbance of stimulated cells/ Mean absorbance of unstimulated cells.

*In vitro Nitric Oxide (NO) production*: To determine the amount of NO production by antigen primed splenocytes collected from experimental mice, a standard Griess assay was performed as per manufacturer instruction (Sigma) [37]. Briefly, single-cell suspension of splenocytes was seeded at a density of 2 × 10^5^ in phenol-red-free complete RPMI 1640 growth medium in 12-well tissue culture plates for 3 h. Next, splenocytes were charged with different concentration of rHcp (0.1 μg/mL, 1.0 μg/mL and 5 μg/mL). After 48 h of incubation, 100 μL of culture supernatant was collected from each well and incubated with an equal volume of Griess reagent (1% sulfanilamide, 0.1% naphthyl ethylenediamine dihydrochloride, 2.5% H_3_PO_4_) at RT for 15 min. The absorbance for each well was measured at 565 nm by using a microplate reader. The nitrite concentration was determined against a standard curve generated with sodium nitrite (NaNO_2_) (Sigma) (Fig. 4a).

*Flow cytometric analysis of T cell phenotypes*: To detect the frequency of T cell subsets, three-color flow cytometry was performed as per the standard method described earlier [38]. Briefly, 1 × 10^6^ splenocytes were suspended in 100 μL of PBS in FACS tube and stained with following monoclonal antibody combinations: CD3-FITC (0.0025 μg/μL), CD4-APC (0.00125 μg/μL) and CD8-PE (0.0025 μg/μL) (eBioscience, Invitrogen) followed by incubation for 30 min at RT in the dark. After incubation, cells were washed with PBS three times and analyzed in BD LSRfortessa flow cytometer (BD Biosciences). The percentage of T cell subsets was selectively gated based on the size and granularity of the cells using the BD FACSDiva software (see Additional file 1: Fig. S2). The T cell phenotypes (CD4^+^ and CD8^+^) were calculated as the percentage of total CD3^+^ T cells.

#### Determination of cecal load of *C. jejuni* in challenged mice

On day 7 post-challenge, experimental mice were sacrificed, and cecum was collected. An equal amount of cecal contents from each mice was weighed, serially diluted in PBS, and plated on *Campylobacter* selectivity agar with CAT supplement. The next day, the colonies that appeared were counted for each experimental group and expressed in log_10_ CFU/gm.

#### In vitro neutralization of *C. jejun*i

To determine the functionality of sIgA induced by the present vaccine composition in vitro neutralization of *C. jejuni* was performed in human INT407 cells as closer mimic outer cell layer of the small intestine. Approximately 1.5 × 10^4^ *C. jejuni* cells were incubated with the fecal soups (undiluted and diluted; 1:10) collected from mice belonging to different experimental groups. After 2 h of incubation, treated *C. jejuni* cells were collected and co-incubated with human INT407 cells at 1:100 MOI for 3 h at 37 °C. After thorough washing, the cells were lysed with 1% triton X-100 (Sigma), serially diluted, and plated onto MH agar plates supplemented with CAT. After incubation overnight under microaerophilic conditions at 37 °C in a tri-gas incubator, the colony appeared were calculated for each experimental group and expressed in log_10_ CFU/mL.

#### Histopathological analysis of cecal tissue

Cecal tissue of the experimental mice challenged with *C. jejuni* were collected at day 7 post last infection and processed for histopathological analysis as per the method described previously [39]. Briefly, 0.5 cm of cecal tissue was first fixed in 10% formal solution, followed by washing under running tap water, and then tissues were dehydrated using the ascending grade of acetone (70%, 90%, and 100%). Tissues were then cleaned with two benzene changes and subsequently impregnated in melted paraffin (62 °C) by three changes of 1 h each. Finally, each paraffin block was sectioned, and the slides were prepared by staining with hematoxylin and eosin (H&E).

### Statistical analysis

Statistical analyses of the data obtained from different assays were analyzed by Graphpad Prism 8.0 software and expressed as the mean ± SE. Shapiro–Wilk test was done to confirm the normal distribution. Comparison between two experimental groups was performed using Student’s t-test (two-tailed, unpaired) or nonparametric Mann − Whitney U test as and when required. P-value less than 0.05 (*P ≤ 0.05) or 0.01 (**P ≤ 0.01) were considered statistically significant.

## Results

### Bioengineered rLAB vector expressing recombinant CWA-Hcp or Sec-Hcp showed stable protein expression

#### Recombinant plasmid encoding *hcp* gene was constructed

The gene sequences of *hcp* were successfully cloned into *L. lactis* based nisin inducible expression cassette of pNZ8048-spUSP45::CWA_M6_ or pSEC-spUSP45 backbone. Restriction digestion and DNA sequencing confirmed the orientation and size of the inserted gene in the respective plasmid constructs. To express Hcp, *L. lactis* (NZ9000) cells were electro-transformed with a recombinant plasmid (pNZ8048-*hcp* or pSEC*-hcp*), and colony PCR confirmed specific amplification of *hcp* gene at the expected size (~ 529 bp) (Fig. 1A-a, b).

#### Nisin inducibility of r*L. lactis* cells were optimized

Analysis of growth profile of un-induced or nisin induced r*L. lactis* secreting or surface expressing Hcp showed an altered growth rate in induced cells (15 ng/mL) compared to the un-induced cells. However, no changes in the growth profile of empty NZ9000 cells were noted following nisin treatment (Fig. 1B-c).

#### Lactococcal surface expression and secretion of Hcp protein was confirmed

**Fig. 2:**
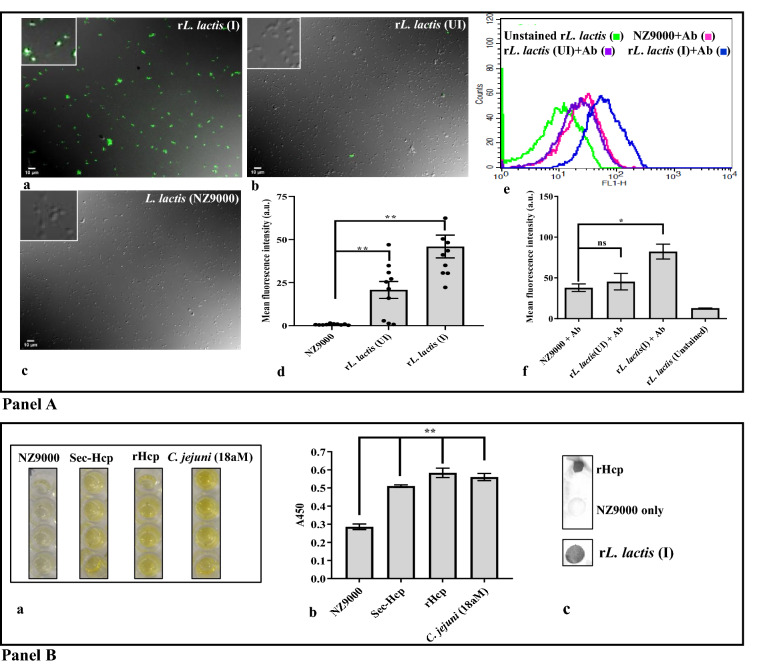
Cell surface expression and secretion of Hcp protein by r*L. lactis.* **A** Representative immunofluorescence images of nisin-induced r*L. lactis* cells surface expressing Hcp probed with rabbit polyclonal anti-Hcp antibody (1:50 dilution), and FITC conjugated goat anti-rabbit IgG secondary antibody (1:500 dilution). Un-induced r*L. lactis* and empty *L. lactis* (NZ000) cells served as control, showing no fluorescence signal. Images were acquired by an inverted fluorescence microscope at 60X magnification. All the images were analyzed using ImageJ software and shown at a scale bar of 10 µm (**a–c**). Data represent mean fluorescence intensity (MFI) of *L. lactis* cells ± SE of three independent experiments. Asterisks indicate a statistically significant increase in the MFI of nisin-induced r*L. lactis* cells (**P ≤ 0.01) with respect to the empty NZ9000 cells (**d**). Flow cytometric analysis of nisin induced *L. lactis* cells showing a significant shift of stained r*L. lactis* population (blue line) as compared to the controls (**e**). Data represent MFI of *L. lactis* cells ± SE of three independent experiments. Asterisks indicate a statistically significant difference (*P ≤ 0.05) compared to the control (**f**). **B** Representative well images indicates specific detection of secreted Hcp in the culture supernatant of induced r*L. lactis* cells harbouring pSEC-spUSP45-*hcp* plasmid by rabbit polyclonal anti-Hcp antibody using indirect ELISA**.** Recombinant Hcp expressed in *E. coli,* and native Hcp secreted by laboratory isolate of *C. jejuni* (18aM) served as a positive control, while empty *L. lactis* cells (NZ9000) were kept as a negative control (**a**). Data represent the mean absorbance (A450) ± SE of quadruplicates samples. Asterisks indicate a statistically significant difference (**P ≤ 0.01) with respect to the control (empty NZ9000) (**b**). Dot blot images showing specific detection of secreted Hcp in the culture supernatant of induced r*L. lactis* cells by rabbit polyclonal anti-Hcp antibody (**c**)

Significantly higher fluorescence intensity of nisin-induced *rL. lactis* cells harbouring pNZ8048-spUSP45-*hcp*-CWA_M6_ plasmid compared to empty or un-induced r*L. lactis* cells confirmed the surface expression of the target protein (**P ≤ 0.01) (Fig. 2A-d).

Further, a clear shift in fluorescence intensity of the gated population of induced r*L. lactis* cells surface expressing Hcp by flowcytometric analysis affirmed the surface localization and accessibility of the expressed protein (*P ≤ 0.05) (Fig. 2A-e, f; Additional file 1: Table S2).

To detect the ability of r*L. lactis* (pSEC-*hcp*) in secreting Hcp, the culture supernatant was subjected for indirect ELISA and immunoblot. Comparative analysis of the signal as detected by ELISA or immunoblot assay indicates the presence of Hcp in the culture supernatant of nisin-induced r*L. lactis* cells (**P ≤ 0.01) (Fig. 2 B-a–c).

### In vivo immunogenicity of mucosal administration of r*L. lactis* in mice

#### Induction of local IgA response (sIgA)

**Fig. 3:**
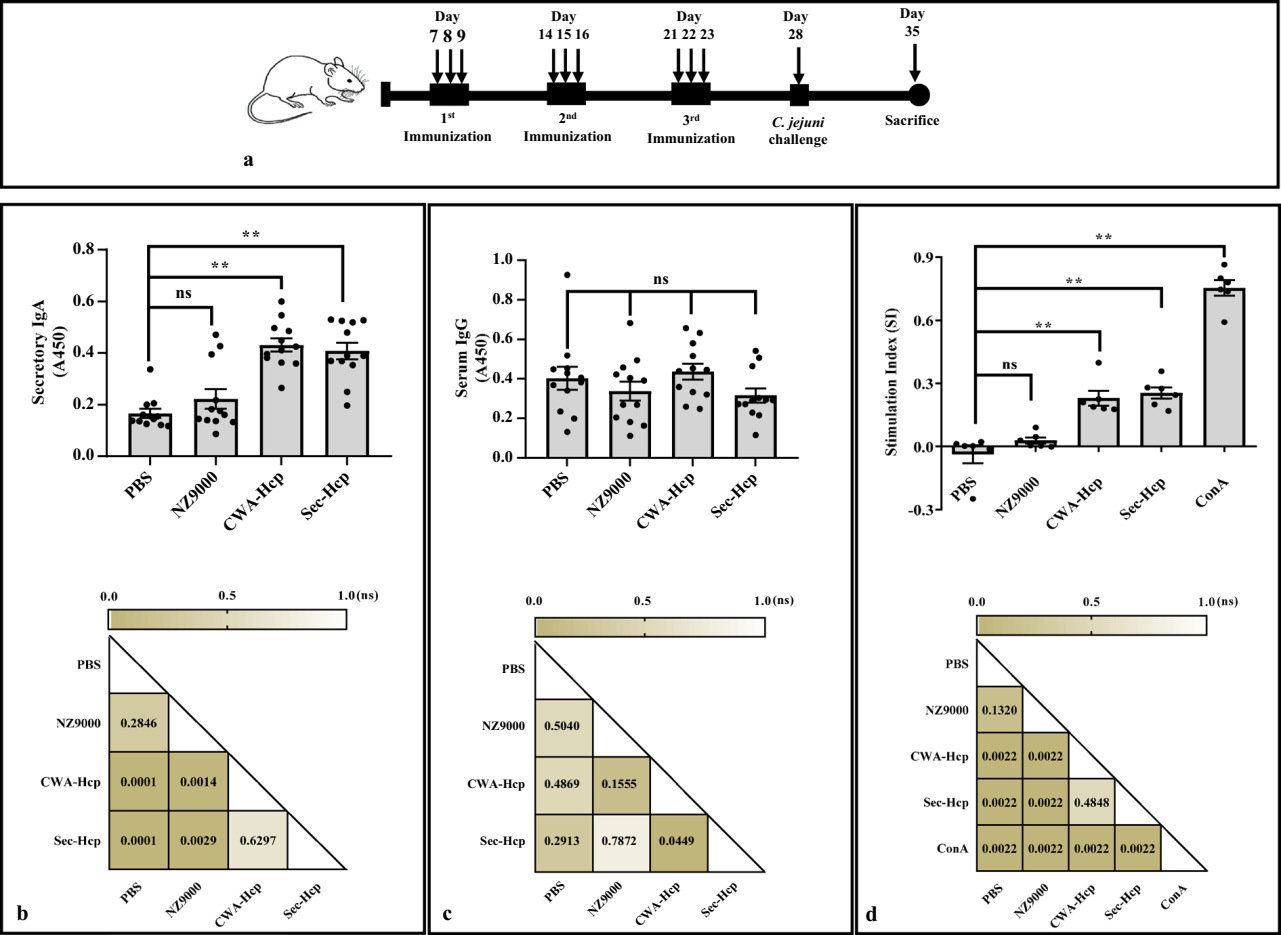
Oral immunization schedule and analysis of local and cellular immune responses in experimental mice. Schematic of the mice immunization schedule**.** On day 7 post last immunization, of ten mice, three mice from each group were sacrificed to obtain spleen, whereas the remaining mice were orally challenged with 2 × 10^9^ CFU of *C. jejuni*. All the challenged mice were sacrificed on day 35 to collect cecal tissue and its contents (**a**). Comparative assessment of Hcp specific local antibody (sIgA) level in fecal soups collected on day 7 post last immunization shows a substantial increment of sIgA level in the groups treated with CWA-Hcp and Sec-Hcp. Each bar represents the mean absorbance (A450) ± SE of 12 mice from three independent experiments (four mice from each experimental setup). Asterisks indicate a statistically significant difference (**P ≤ 0.01) compared to the control group (PBS only) (**b**). However, no such change was observed in the serum IgG level among the groups. ‘ns’ represents a non-significant difference between different experimental groups. (**c**) Splenocytes collected from different experimental groups were stimulated with 10 μg/mL of rHcp. For each experiment, a total of three animals were randomly selected from two independent experiments performed under similar conditions (n = 6). Each assay was performed in triplicates, and the absorbance was measured at 595 nm. The bar represents the mean stimulation index ± SE of six mice. Asterisks indicate a statistically significant difference in the treated group (**P ≤ 0.01) with respect to the control group (PBS only) (**d**). A pairwise statistical comparison among the groups was presented using a “P-value matrix” (**b**, **c**, **d**). The darker boxes represent the P-value closer to 0.01 (significant), while the lighter boxes represent P-value closer to 1 (non-significant)

To test the ability of mucosal delivery of r*L. lactis* expressing Hcp in inducing antigen-specific local antibody response, fecal soups and sera samples collected on day 7 post last immunization were analyzed for sIgA and serum IgG response respectively. Comparative analysis suggests that regardless of the protein localization, both forms of r*L. lactis* were able to induce high-level sIgA production in the intestine as compared to the control mice (immunized vs. control) (**P ≤ 0.01) (Fig. 3b). However, in terms of serum IgG response, no such changes were observed (Fig. 3c).

### Enhancement of Hcp specific cell-mediated immune responses

#### Stimulation of antigen-specific splenic lymphocytes response

The Hcp specific splenocyte proliferation was found to be significantly higher in the mice that received either cell wall anchored or secretory form of Hcp compared to the control groups (PBS or NZ9000) (**P ≤ 0.01) (immunized vs. control) (Fig. 3d).

#### Significant increase in nitric oxide (NO) production

**Fig. 4:**
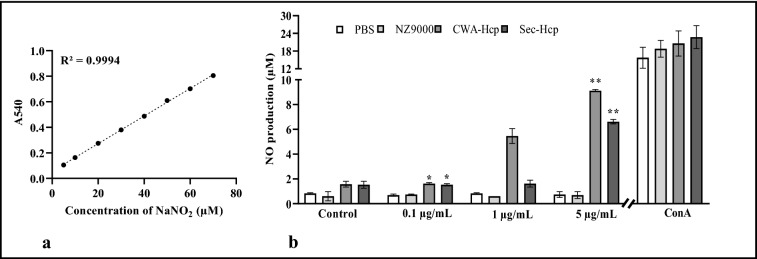
Nitric oxide (NO) production by splenocyes. The nitrite concentration in the supernatant of splenocytses culture was determined against sodium nitrite (NaNO_2_) as a standard. For this first a standard curve of nitrite concentration (x-axis) against absorbance (y-axis) was plotted (**a**). At day 7 post last immunization, production of NO was determined by adding Griess reagent in the culture supernatant of splenocytes collected from different experimental mice and stimulated with varying concentrations of rHcp (0.1 μg/mL, 1 μg/mL, and 5 μg/mL). Concanavalin A (ConA) served as a positive control. For each experiment, a total of three mice were randomly selected from two independent experiments performed under similar conditions (n = 6). Data represent the mean NO production ± SE of six mice from two independent experiments. Asterisks indicate a statistically significant difference (*P ≤ 0.05, **P ≤ 0.01) compared to the control (received PBS only) (**b**)

In response to in vitro stimulation with different concentrations of rHcp, high-level production of NO was detected in the culture supernatants of splenocytes collected from immunized mice (CWA-Hcp and Sec-Hcp) (*P ≤ 0.05; **P ≤ 0.01) (immunized vs. control) (Fig. 4b). However, only a basal level of NO production was noted in the case of the mice treated with empty NZ9000 cells compared to the unimmunized group of mice (received PBS only).

#### Change in T cell phenotype population (CD4^+^**and CD8**^+^)

**Fig. 5:**
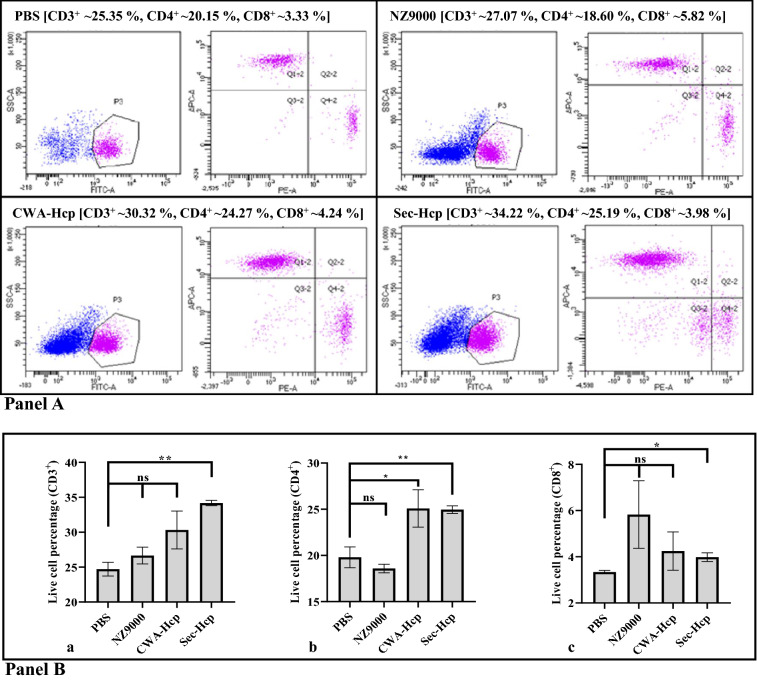
Immunophenotyping of T cell subsets by flow cytometry. **A** Splenocytes collected at day 7 post last immunization were stained with CD3-FITC, CD4-APC, and CD8-PE monoclonal antibodies. Splenocytes were gated based on their FSC/SSC to remove the dead cells, while FSC-H vs. FSC-A density plots was used to exclude doublets (see Additional file 1: Fig. S2). Flow cytometric analysis was performed with a gated population of T cells (FITC), Th cells (APC), and Tc cells (PE) collected from various experimental groups. For immunophenotypic profiles of Th and Tc cells, triple staining was performed (CD3-FITC, CD4-APC, and CD8-PE). Channels FL1 and FL2 were used as filters or detectors for FITC and PE-labelled antibodies, respectively. **B** Live cell percentage of gated T cells and other subsets (Th and Tc) among different experimental groups. For each experiment, a total of three animals was randomly selected from two independent experiments performed under similar conditions (n = 6). The mean percentage (%) of live T cells population in splenocytes obtained from the mice of each experimental group showed a minor increment in T cell (CD3^+^) population in case CWA-Hcp group while a significant increase was observed in the group of mice that received r*L. lactis* expressing Sec-Hcp (**P ≤ 0.01) compared to the control group of mice those received PBS only (**a**). A significant increment of the mean live T (CD4^+^) cell percentage in the different experimental groups (CWA-Hcp, *P ≤ 0.05; Sec-Hcp, **P ≤ 0.01) was observed (**b**). The mean live T (CD8^+^) cell percentage in splenocytes obtained from the mice that received Sec-Hcp showed a significant increment (*P ≤ 0.05) in the T cell population compared to the control mice those received PBS only (**c**). Data represent live cell percentage ± SE of two independent experiments. Asterisks indicate a statistically significant difference (*P ≤ 0.05, **P ≤ 0.01) with respect to the control group (received PBS only)

Compared to the control groups, a minor increase in the total T cell population (CD3^+^) was observed in the mice that received r*L. lactis* expressing CWA-Hcp (∼30.32%) (Additional file 1: Table S1). In contrast, a significant increase was noted in the case of Sec-Hcp (∼34.22%) (**P ≤ 0.01) as compared to the control group of mice (PBS ∼25.35% or empty NZ9000 cells ∼27.07%) (Fig. 5B-a). The critical analysis of subsets of T cells further suggest a significant rise in CD4^+^ T cells in both the immunized groups (CWA-Hcp, *P ≤ 0.05; Sec-Hcp, **P ≤ 0.01) while for CD8^+^ T cell population, mice that received Sec-Hcp only showed significant increment (*P ≤ 0.05) compared to PBS group (Fig. 5B-b, c).

### Reduction of the cecal load of *C. jejuni* in mice administered with rLAB vectors

**Fig. 6:**
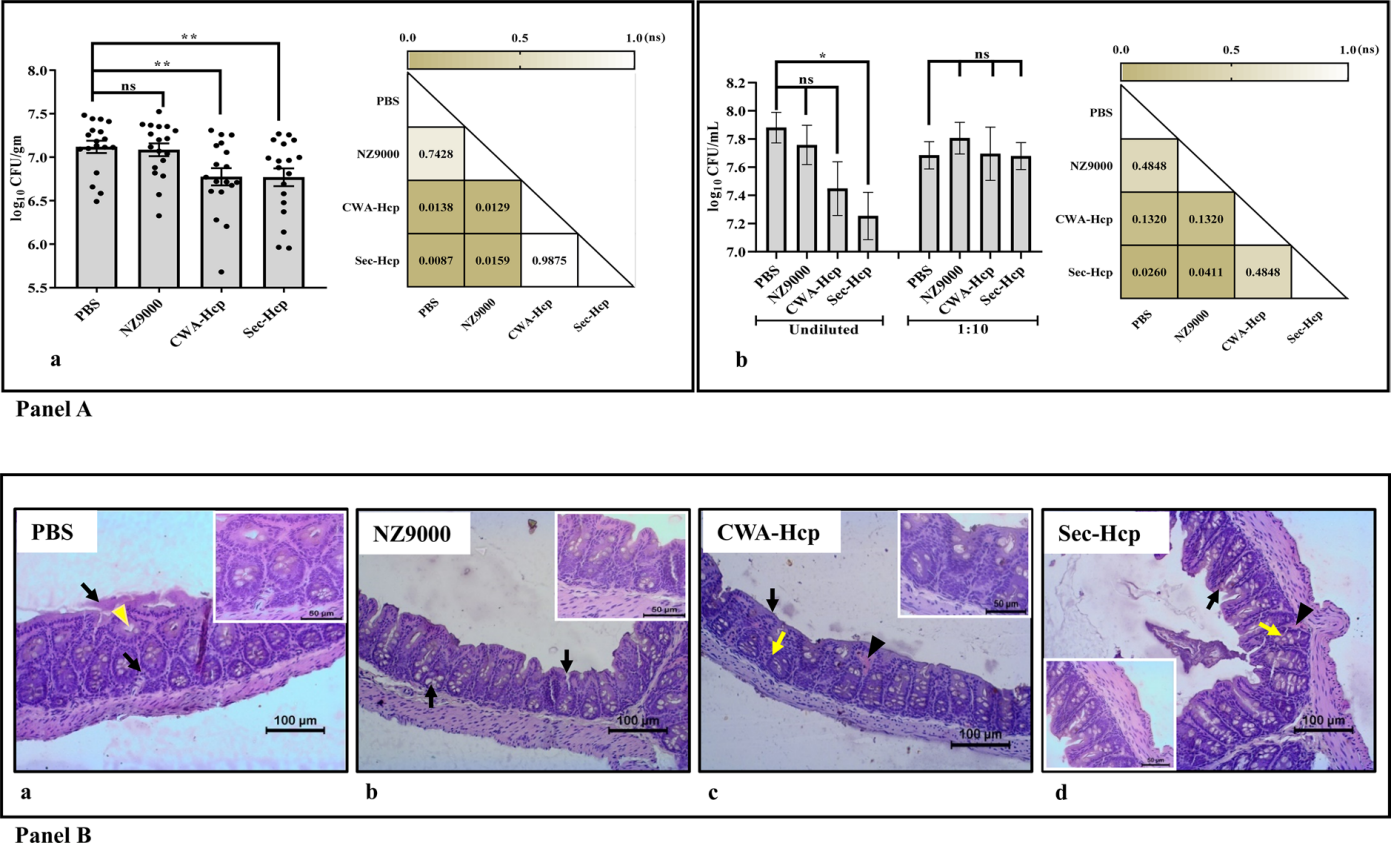
Protective efficacy and histopathological analysis of experimental mice challenged with *C. jejuni.* **A** Data showed a significant reduction in the cecal load of *C. jejuni* in mice that received r*L. lactis* express either cell wall-anchored or secretory form of Hcp compared to the control groups (PBS or NZ9000 only). For each experiment, total of six mice were sacrificed from three independent experiments performed under similar conditions. Data represent mean log_10_ CFU/gm ± SE of three independent experiments (n = 18). Asterisks indicate a statistically significant difference compared to PBS control group (**P ≤ 0.01) (**a**). In vitro blocking of *C. jejuni* adhesion and invasion to human INT407 cells by antigen-specific neutralizing antibody (sIgA) present in the fecal soups of the immunized group (Sec-Hcp) of mice (*P ≤ 0.05; immunized vs. control). Data represent mean log_10_ CFU/mL ± SE of three mice (n = 6) from two independent experiments (**b**). A pairwise statistical comparison among the groups was presented using a “P-value matrix” (**a**, **b**). The darker boxes represent the P- value closer to 0.01 (significant), while the lighter boxes represent P-value closer to 1 (non-significant). **B** Histological examination of formalin-fixed paraffin-embedded sections of the cecal tissue collected from unimmunized mice (PBS only) at day 7 post-infection with *C. jejuni*. Section of cecal tissue showed significant mucosal hyperplasia exhibited by extensive luminal erosion of the epithelial lining, loss of villi, and infiltration of polymorphonuclear cells (black arrow). *C. jejuni* characterized by spiral morphology was detected in the enterocyte of superficial mucosa (yellow arrowhead). Inset: Magnified image (**a**). Section of cecal tissue from mice that received empty *L. lactis* (NZ9000) cells showing focal necrosis in the lining epithelial and glandular cells (black arrow) (**b**). In case mice that received mucosal administration of r*L. lactis* expressing Hcp, show evenly organized epithelial cells in a single layer **(**black arrow). The epithelial cell population in the gland shows a tightly arranged cell structure (yellow arrow). Focal accumulation of eosinophilic proteinaceous material in submucosa in both immunized groups indicates the possible presence of secretory antibodies (arrowhead) (**c**). Presence of eosinophilic non-inflammatory material beneath the superficial enterocyte lining (arrowhead). The number of goblet cells was more in the Sec-Hcp group of mice (black arrow) (**d**)

To evaluate the effect of mucosal administration of r*L.lactis* expressing Hcp, the cecal load of each mice was calculated on day 7 post-challenge with *C. jejuni*. Present data suggest reduced bacterial load in the cecum of the immunized mice compared to the control (immunized vs. control) (**P ≤ 0.01) (Fig. 6A-a).

### In vitro neutralization of *C. jejuni* by local antibody (sIgA)

Direct comparison of total CFU of *C. jejuni* present in infected human INT407 cells suggest significant inhibition of host cells adhesion and invasion of *C. jejuni* by the sIgA present in the intestine of Hcp-immunized mice as compared to the controls (immunized vs. control) (*P ≤ 0.05) (Fig. 6 A-b; Additional file 1: Table S3).

### Histopathological finding in cecal tissue

The comparative histopathology of cecal tissue sections collected from different experimental groups of mice showed fewer lesions in the CWA-Hcp (Fig. 6B-c) and Sec-Hcp (Fig. 6B-d) administered groups compared to the control groups (PBS, Fig. 6B-a; or NZ9000, Fig. 6B-b). Specifically, group of mice that received r*L. lactis* expressing CWA-Hcp exhibits the normal architecture of the cecal lumen characterized by properly arranged epithelial cell linings in a single layer without any erosion or inflammatory change (black arrow). Tightly arranged epithelial cell population in the glands also suggest effective protection of intestinal mucosa against *C. jejuni* infection.

In xcontrast, mice administered with r*L. lactis* expressing Sec-Hcp, except for some diminutive necrotic lesions in the lining of cecal epithelial cells, no other pathological change was noticed (Fig. 6B-d).

Cecum sections collected from the mice administered with PBS or NZ9000 showed infection-induced marked tissue damages exhibited by loss of villi in the epithelial cells. Moreover, moderate to a high degree of mucosal hyperplasia with clear luminal erosion of epithelial lining and a significant number of focal necrotic lesions were evident in the tissue sections. Notably, spiral-shaped foreign bodies in the enterocyte of superficial mucosa indicates the intracellular location of *C. jejuni* (yellow arrowhead)*.*

## Discussion

*Campylobacter* utilizes several putative virulence factors, including an organized secretion system to evade the host defense and facilitates bacterial pathogenesis. Specifically, the role of major effector proteins of recently discovered T6SS of *C. jejuni* in bacterial self-survival, host cell attachment, subsequent pathogenesis, and niche establishment have prompted us to explore the potential of a T6SS targeted vaccine against *C. jejuni* [40, 41].

Of the 13 core proteins of complete T6SS, Hcp is recognized as a critical protein essential for T6SS assembly and its effector functions [30]. Given these unique structural and functional characteristics of Hcp, recently, we showed the advantage of mucosal administration of rHcp in conferring immune-protection against *C. jejuni* in the chicken model; however, it failed to show any cecal pathology [22, 31]. Although chickens remain the most implicated source for *C. jejuni* transmission to humans, it maintains a commensal relationship with little or no pathogenicity in the avian gut, particularly in the crypts of the caeca [42]. Despite high-level Hcp-specific local immune responses in the chickens gut, moderate immune-protection in terms of cecal load of *C. jejuni* is possibly attributed to its commensal nature. Moreover, human campylobacteriosis exhibits a different set of clinical outcomes from the chickens hosts. Therefore, to investigate vaccine-induced immune-protection for humans against *C. jejuni* infection needs a more reliable and reproducible model to study. To this end, the present study was undertaken to evaluate the effect of Hcp immunization in reducing the intestinal load of *C. jejuni* using an inbred normal flora mice model.

Because the mucosa and its associated lymphoid tissues play a central role in the host defense against gut pathogens, including *C. jejuni*, we specifically chose the mucosal route to deliver the target antigen using a bioengineered LAB vector. To achieve this, first, a food-grade LAB, *L. lactis* was engineered to express Hcp protein of *C. jejuni* T6SS. The majority of the Hcp family proteins (Hcp1 and Hcp2) are typically anchored in the bacterial outer membrane and facilitate the assembly of a functional T6SS [43]. However, Hcp1 is primarily secreted in a T6SS-dependent manner to induce actin cytoskeleton rearrangement, apoptosis, and cytokine release [43]. Unlike other Gram-negative bacteria, regardless of serotypes, *C. jejuni* T6SS possesses only one Hcp gene purported to play both functions [41]. Therefore, to achieve native structural and functional activity of *C. jejuni* Hcp, two different food-grade *L. lactis* were constructed, one expressing Hcp on the surface and the other secreting Hcp directly in the culture medium. In addition, a sub-optimal concentration of nisin was applied to express Hcp protein using NICE system driven by P_*nisA*_ promoter to evoke an efficient but controlled expression of the target protein by bioengineered LAB vectors. Consistent with our hypothesis, we showed specific detection of Hcp either on the cell surface or in the culture supernatant of optimally induced r*L. lactis* cells using an anti-Hcp polyclonal antibody raised in rabbits. These observations also endorsed the ability of rLAB vector in expressing the immunologically relevant protein of *C. jejuni* T6SS.

In the next, a head-to-head comparison of *rL. lactis* expressing Hcp was performed to study the immune phenotypes for each type of vaccine modality. We assessed the magnitude and the quality of the local and systemic immune responses imparted by a prime-boost vaccination strategy in mice. Our data suggest that both modalities induced a strong local sIgA response while in vitro neutralization of *C. jejuni* using fecal soups from immunized mice confirmed its functionality of local antibodies [22]. Similar to other gut pathogens, initial clearance of extracellular *C. jejuni* is expected to be largely dependent on opsonophagocytosis facilitated by antigen-specific antibodies [44]. We showed the ability of Hcp-specific local sIgA to neutralize and quench *C. jejuni* adherence to human INT407 cells used as closer mimic non-polarised enteric cells [45, 46]. In line with our previous study, the observed neutralization of *C. jejuni* also suggests that local sIgA antibodies might be crucial for blocking T6SS mediated binding and neutralizing *C. jejuni* while it is in the extracellular stage. In addition, our histological analysis of cecal tissue indicates the intracellular existence of *C. jejuni,* particularly in the enterocyte of the superficial mucosal epithelium.

Together with effective neutralization of extracellular *C. jejuni,* a strong cellular immune response is critical [47]. To this end, our immunophenotyping data submit the ability of mucosally delivered Hcp in modest increment of both CD4^+^ and CD8^+^ T cell population. Moreover, splenocytes collected from the immunized mice showed a robust proliferative response with high-level production of NO upon stimulation with Hcp antigen. As a critical immune effector function of activated CD4^+^ T cell response, the presence of high-level NO in the culture supernatant of Hcp primed T cells suggests the ability of the current vaccine modality in activation of IFN-γ signaling pathway to upregulate inducible nitric oxide synthase (iNOS) [48]. Together, the current set of data suggest the capacity of both forms of rLAB vector in presenting carrier antigen to the T cells via MHC class I and class II-restricted pathway to provide all-round protection against *C. jejuni* and other gut pathogens [49, 50].

It is of particular interest to see whether immunization with rLAB vector expressing Hcp could protect the gut mucosa from *C. jejuni* pathogenesis. In line with our previous findings, the present study further reflects the reproducibility for some immuno-pathological outcomes in the mice model of *C. jejuni* infection [39]. Particularly as an important observation, we report that infection-induced changes in cecal tissue of unimmunized control mice show exacerbated inflammatory changes characterized by significant loss of villi, focal necrotic lesions in epithelial cells, while in the case of immunized mice, such changes were found to be substantially low. However, for precise demonstration of mirroring the exact clinicopathological outcomes of *C. jejuni* infection in humans, more advanced animal models such as “gnotobiotic” and ‘‘humanized’’ mice may result in full scale immunological correlates of protection against *C. jejuni* infection [50–55]. Together with the significant reduction in cecal load of *C. jejuni* without cell cytopathy, strong local and systemic immune responses in immunized mice support the ability of the present vaccine composition to protect intestinal mucosa from *C. jejuni* pathogenesis and conferring immune-protection against *C. jejuni* colonization.

Despite these advantages, the observed reduction in cecal load of *C. jejuni* was found to be low compared to oral immunization strategy where attenuated *Salmonella enterica* or *Lactobacillus* spp. was employed. This could presumably result from their longer transit time or gut colonizing nature, leading to the mounting of effective antigen presentation to the local immune cells [56, 57]. Moreover, it should be noted that the genetic manipulation of LAB vector, though represents a safer alternative to adjuvanted vaccine delivery strategy, both directed and uncontrolled genetic modifications of recombinant *L. lactis* are classified under the Genetically Modified Organism (GMO) category [58, 59].

Moving forward with this work, as a Generally Recognized as Safe (GRAS) category non-invasive bacteria, since *L. lactis* exhibits shorter gut transit time, a better understanding of the bio-availability of protein expressed by rLAB vector is critical to achieve optimal antigen presentation in the gut.

## Conclusion

Together with its intrinsic adjuvanticity, our preliminary data highlights the benefit of the present vaccine approach in maintaining gut homeostasis and protecting intestinal mucosa from *C. jejuni* pathogenesis without the risk of immune-tolerance. In particular, given their ability to stimulate both the mucosal and systemic immune system by controlled expression of target protein, we submit that bioengineered LAB vector could be considered as a promising mucosal vaccine approach against a common gut pathogen, including *C. jejuni*. Since the success of a mucosal vaccine candidate largely depends on controlling bacterial adhesion and invasion of the gut epithelium, additional measure to drive the optimal expression of the target protein is critically important to ensure versatile use of LAB vector.

## Supplementary Information

**Additional file 1: Table S1.** Percentages of different T cell populations (CD3^+^, CD4^+^ and CD8^+^) of experimental mice. **Table 2.** Mean fluorescence intensity (MFI) of empty *L. lactis* (NZ9000), un-induced or nisin induced *rL. lactis* cells surface expressing rHcp; **Table S3.** In vitro neutralization assay: Number of *C. jejuni* associated with human INT407 cells; **Fig. S1.** Cytotoxic effect of rHcp secreted by *L. lactis* (Sec-Hcp) in human INT407 cells; **Fig. S2.** Gating strategy of the T cell population for immunophenotyping.

## Data Availability

All data generated or analyzed during this study are included in this published article [and its additional files].
