# Supplementary material for: Bioengineering of LAB vector expressing Haemolysin co-regulated protein (Hcp): a strategic approach to control gut colonization of Campylobacter jejuni in a murine model
Source: Gut Pathog. 2021 Jul 30;13:48. doi: 10.1186/s13099-021-00444-2 (PMC8323230; doi:10.1186/s13099-021-00444-2)
Supplement: Supplementary file 1 — Additional file 1: Table S1. Percentages of different T cell populations (CD3+, CD4+ and CD8+) of experimental mice. Table 2. Mean fluorescence intensity (MFI) of empty L. lactis (NZ9000), un-induced or nisin induced rL. lactis cells surface expressing rHcp; Table S3. In vitro neutralization assay: Number of C. jejuni associated with human INT407 cells; Fig. S1. Cytotoxic effect of rHcp secreted by L. lactis (Sec-Hcp) in human INT407 cells; Fig. S2. Gating strategy of the T cell population for immunophenotyping. [file 13099_2021_444_MOESM1_ESM.pdf]

Additional file

Bioengineering of LAB vector expressing Haemolysin co-regulated protein (Hcp): a strategic approach to control gut colonization of C*ampylobacter jejuni* in murine model

Chandan Gorain[a], Afruja Khan[a], Ankita Singh[a], Samiran Mondal[b] and Amirul Islam Mallick[a]*

[a] Department of Biological Sciences, Indian Institute of Science Education and Research Kolkata, Mohanpur, Nadia, West Bengal-741246, India

[b] Department of Veterinary Pathology, West Bengal University of Animal and Fishery Sciences, Belgachia, Kolkata, West Bengal-700037, India

*Corresponding Author

Corresponding Author's address:

Dr. Amirul Islam Mallick, Associate Professor, Department of Biological Sciences, Indian Institute of Science Education and Research Kolkata, Mohanpur, Nadia, West Bengal, 741246, India. Phone: +91 033 2502 8000 (Ext 1221) Fax: +91-33-2502 8002.

E-mail amallick@iiserkol.ac.in

Table S1: Percentages of different T cell populations ($CD3^+$, $CD4^+$ and $CD8^+$) of experimental mice

Experimental groups (n=6)	Live cell percentage ± SE		
	T cells ($CD3^+$)	Th cells ($CD4^+$)	Tc cells ($CD8^+$)
PBS	25.35 ± 1.274	20.151 ± 1.45	3.336 ± 0.056
Empty NZ9000	27.075 ± 1.296	18.601 ± 0.534	5.829 ± 1.133
CWA-Hcp	30.32 ± 2.711	24.272 ± 2.278*	4.249 ± 0.829
Sec-Hcp	34.225 ± 0.429**	25.193 ± 0.388**	3.981 ± 0.168*

Data represent the mean percentage of total T cells and other subsets (Th and Tc) among different experimental groups (*$P \leq 0.05$, **$P \leq 0.01$; immunized vs. control).

Table S2: Mean fluorescence intensity (MFI) of empty *L. lactis* (NZ9000), un-induced or nisin induced r*L. lactis* cells surface expressing rHcp

Experimental groups	r*L. lactis* (Unstained)	NZ9000 + Ab	r*L. lactis* (UI) + Ab	r*L. lactis* (I) + Ab
**Mean fluorescence intensity (a.u.) ± SE**	12.86 ± 0.251	37.94 ± 4.633	45.37 ± 10.174	82.27 ± 9.134*

Data represent the mean fluorescence intensity (MFI) ± SE of three independent experiments (*$P \leq 0.05$, NZ9000 vs. Induced).

Table S3: *In vitro* neutralization assay: Number of *C. jejuni* associated with human INT407 cells.

**Experimental groups (n=6)**		**Mean log_{10} CFU/mL ± SE**
Undiluted	PBS	7.741 ± 0.173
	Empty NZ9000	7.757 ± 0.139
	CWA-Hcp	7.606 ± 0.213
	Sec-Hcp	7.411 ± 0.193*
1:10 dilution	PBS	7.684 ± 0.096
	Empty NZ9000	7.806 ± 0.112
	CWA-Hcp	7.695 ± 0.188
	Sec-Hcp	7.678 ± 0.095

Data indicate the number of *C. jejuni* (log_{10} CFU/mL of *C. jejuni* ± SE) associated with (adhered +invaded) human INT497 cells. The experiment was performed twice under similar condition (**P* ≤0.05, PBS vs. treatment).

Assessing the cytotoxicity of rHcp secreted by r*L. lactis* (Sec-Hcp)

To determine the cytotoxicity of r*L. lactis* secreting Hcp, MTT based cell viability assay was performed. Briefly, human INT407 cells were seeded at a density of 4 x 10^5 cells per well in the lower chamber of a 24 well transwell plate system (Merck) in a complete DMEM medium and incubated at 37 °C under 5% CO_2 pressure in a humidified environment till 90% confluency. Next, r*L. Lactis* cells (Sec-Hcp) were grown in M17 medium supplemented with 0.5% glucose and 20 µg/mL chloramphenicol till OD_{600} reached 0.3. Following, 1 x 10^9 bacterial cells were added in the upper chamber of the trans well system provided with 15ng/mL of nisin and incubated for another 4 h at 30 °C. After incubation, the bacterial cells were removed from the transwell system and the plate containing the bacterial supernatant was further incubated for 12 h at 37 °C. Following incubation, INT407 cells were washed with PBS and fresh complete media was added. Finally, to each well, 30 µL of MTT (3-[4, 5-dimethylthiazolyl-2]-2, 5-diphenyl tetrazolium bromide) dye (1 mg/ml) was added and incubated for another 3 h at 37 °C under 5% CO_2 pressure. The water-insoluble formazan

crystals thus formed were solubilized with dimethyl sulphoxide (DMSO) (500 µL/well), and the absorbance was measured at 595 nm using a microplate reader. Untreated human INT407 cells and cells treated with empty *L. lactis* (NZ9000) were kept as control (Fig. S1).

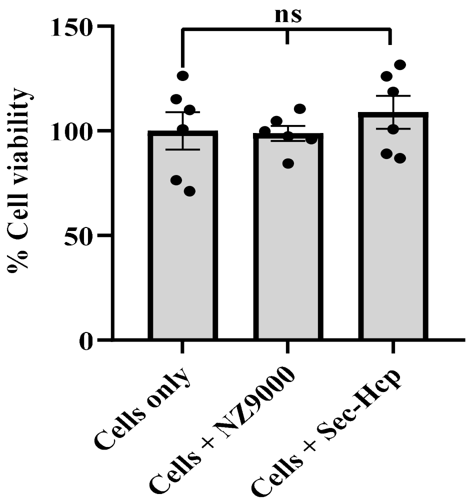

Fig. S1: Cytotoxic effect of rHcp secreted by *L. lactis* (Sec-Hcp) in human INT407 cells. The cytotoxicity of r*L. lactis* expressing Hcp was determined by standard MTT assay. INT407 cells, seeded in a 24 well plate transwell system, were treated with nisin-induced r*L. lactis* (Sec-Hcp) and incubated for 4 h at 30 °C. After incubation, bacterial cells were removed, followed by incubation at 37 °C for additional 12 h. Next, the INT407 cells were washed with PBS and replaced with fresh complete medium. Finally, 30 µL of MTT was added to each well and incubated for next 3 h. Formazan crystal thus formed were dissolved in DMSO. The absorbance was read at 595 nm. Data confirm the non-toxic nature of rLAB vector to human INT407 cells exhibited by little or no cell cytotoxicity. Individual dots represent the mean cell viability ± SE compared to untreated cells normalized to 100 %..

Gating strategy of the T cell population for immunophenotyping

To analyse the subsets of T cell population, three-color flow cytometry was performed. The splenocytes obtained from different experimental groups of mice were stained with the following monoclonal antibody combinations: CD3-FITC (0.0025 μg/μL), CD4-APC (0.00125 μg/μL) and CD8-PE (0.0025 μg/μL) (eBioscience, Invitrogen) followed by incubation for 30 min at RT in the dark. After incubation, cells were washed with PBS three times and analyzed in BD LSRfortessa flow cytometer (BD Biosciences). Gating strategy for the analyses and sorting of isolated splenocytes from mice administered with r*L. lactis* was performed using FSC/SSC plots. The percentage of T cell subsets was selectively gated based on the size and granularity of the cells using the BD FACSDiva software. Dead Cells were removed based on forward (FSC) and side scatters (SSC), while aggregates and doublet cells were excluded using scatter areas versus their respective width parameters FSC-H vs. FSC-A.

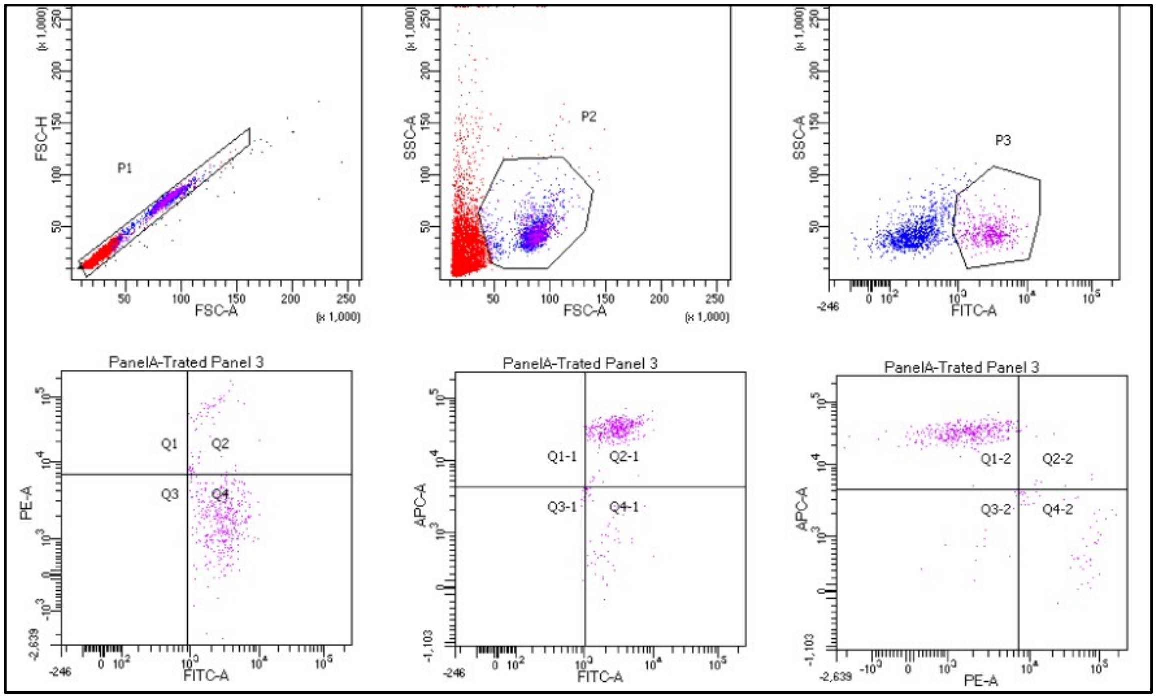

Group A: PBS

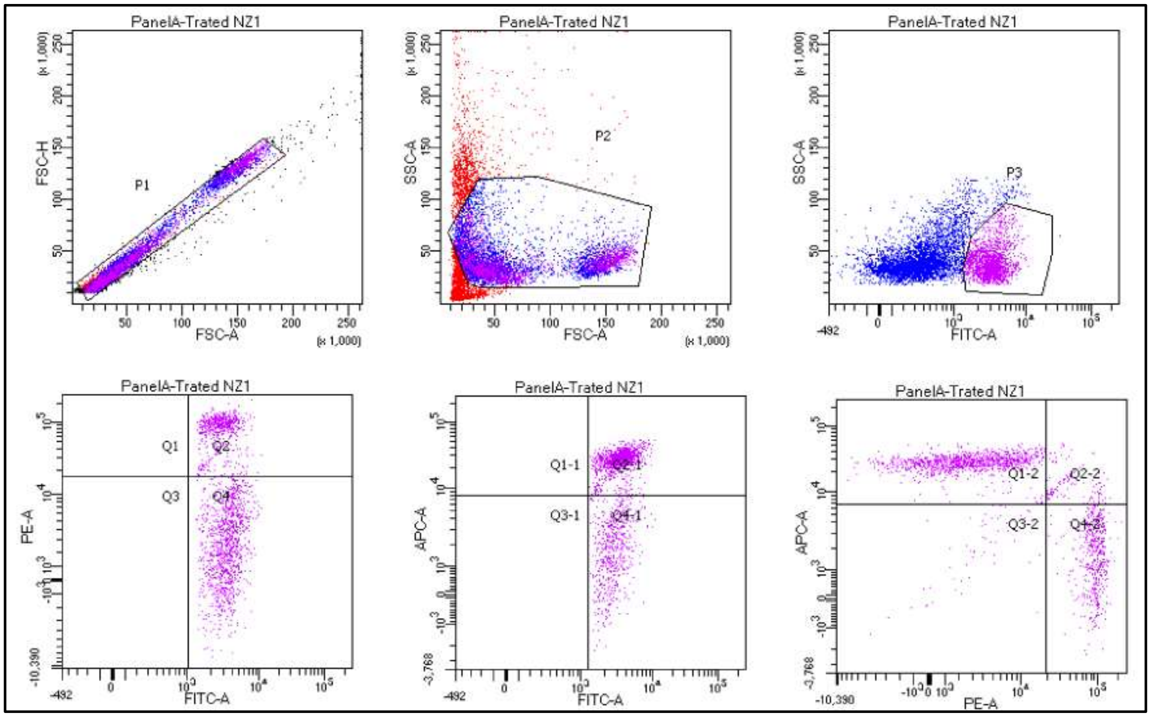

Group B: NZ9000

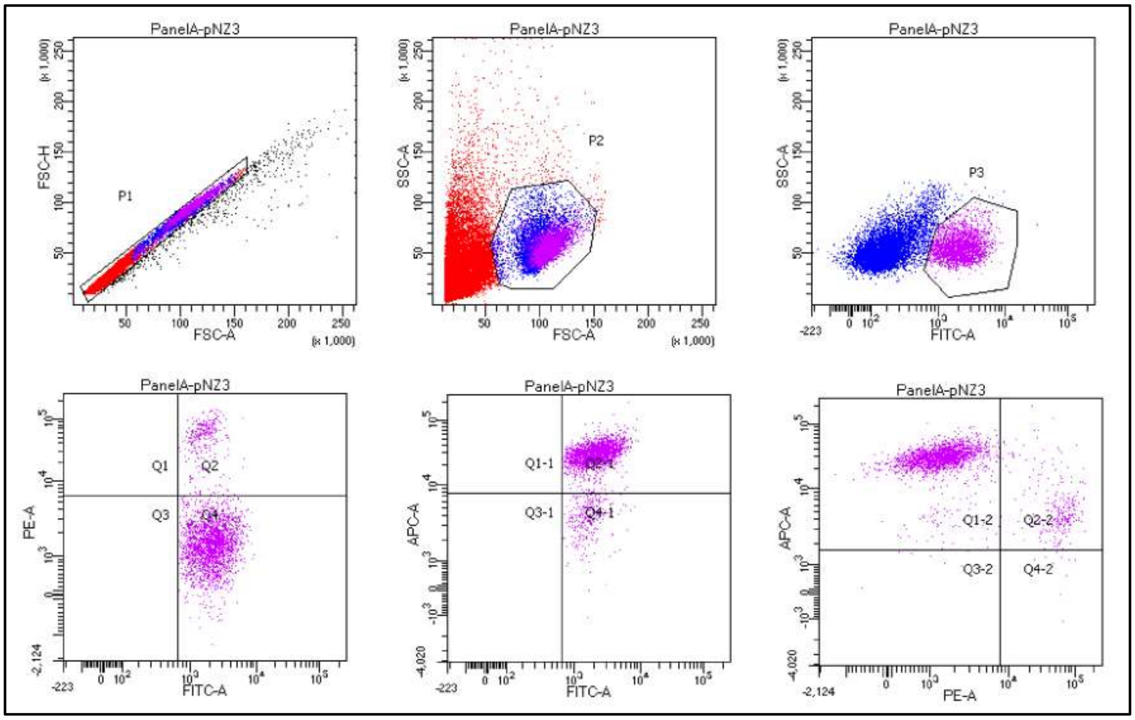

Group C: CWA-Hcp

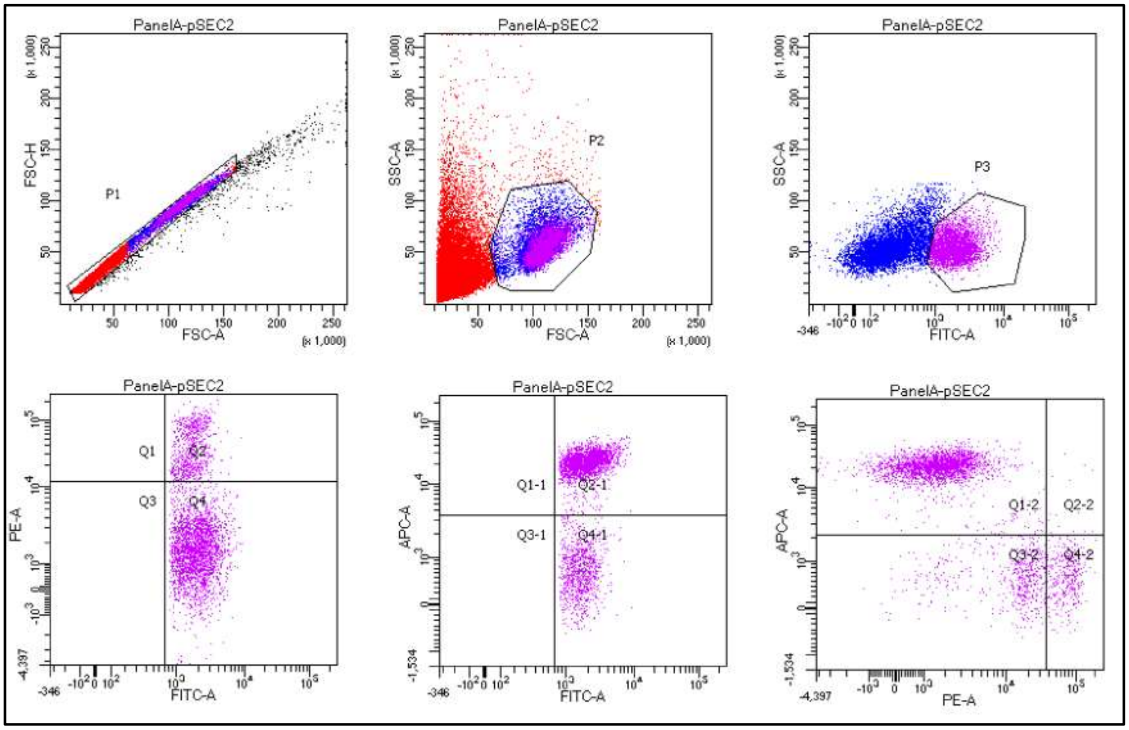

Group D: Sec-Hcp

Fig. S2: Representative FSC/SSC plot of Tcell population for each experimental group of mice.

A FSC vs. SSC plot showing total cell population (Left upper corner). Dead Cells were removed based on forward scatter (FSC) and side scatter (SSC), while aggregates and doublet cells were excluded using scatter areas versus their respective width parameters (FSC-H vs. FSC-A). The black circle highlights the total live cell population (upper middle). Next, gated cells (pink color; upper right corner) indicating T cell population based on expression of $CD3^+$ (FITC labelled). Out of the total T cells, subsets of T cell population (Th and Tc cells) were sorted using APC and PE monoclonal antibodies. The lower-left corner gate is indicating T cytotoxic cell population based on $CD8^+$ expression, while the lower middle gate is showing T helper cell population based on $CD4^+$ expression. APC-A vs. PE-A represents the percentage of $CD4^+$ and $CD8^+$ cell population respectively (lower right corner).
